# The Four-Way Stop Sign: Viruses, 12-Lipoxygenase, Islets, and Natural Killer Cells in Type 1 Diabetes Progression

**DOI:** 10.3389/fendo.2017.00246

**Published:** 2017-09-25

**Authors:** Michele L. Semeraro, Lindsey M. Glenn, Margaret A. Morris

**Affiliations:** ^1^Department of Internal Medicine, Strelitz Diabetes Center, Eastern Virginia Medical School, Norfolk, VA, United States

**Keywords:** coxsackievirus infections, islets, natural killer cells, 12-lipoxygenase, type 1 diabetes

## Abstract

Natural killer (NK) cells represent an important effector arm against viral infection, and mounting evidence suggests that viral infection plays a role in the development of type 1 diabetes (T1D) in at least a portion of patients. NK cells recognize their target cells through a delicate balance of inhibitory and stimulatory receptors on their surface. If unbalanced, NK cells have great potential to wreak havoc in the pancreas due to the beta cell expression of the as-yet-defined NKp46 ligand through interactions with the activating NKp46 receptor found on the surface of most NK cells. Blocking interactions between NKp46 and its ligand protects mice from STZ-induced diabetes, but differential expression non-diabetic and diabetic donor samples have not been tested. Additional studies have shown that peripheral blood NK cells from human T1D patients have altered phenotypes that reduce the lytic and functional ability of the NK cells. Investigations of humanT1D pancreas tissues have indicated that the presence of NK cells may be beneficial despite their infrequent detection. In non-obese diabetic (NOD) mice, we have noted that NK cells express high levels of the proinflammatory mediator 12/15-lipoxygenase (12/15-LO), and decreased levels of stimulatory receptors. Conversely, NK cells of 12/15-LO deficient NOD mice, which are protected from diabetes development, express significantly higher levels of stimulatory receptors. Furthermore, the human NK92 cell line expresses the ALOX12 protein [human 12-lipoxygenase (12-LO), related to mouse 12/15-LO] *via* Western blotting. Human 12-LO is upregulated in the pancreas of both T1D and T2D human donors with insulin-containing islets, showing a link between 12-LO expression and diabetes progression. Therefore, our hypothesis is that NK cells in those susceptible to developing T1D are unable to function properly during viral infections of pancreatic beta cells due to increased 12-LO expression and activation, which contributes to increased interferon-gamma production and an imbalance in activating and inhibitory NK cell receptors, and may contribute to downstream autoimmune T cell responses. The work presented here outlines evidence from our lab, as well as published literature, supporting our hypothesis, including novel data.

## Introduction

Autoimmune destruction of the pancreatic beta cells leads to the development of Type 1 diabetes (T1D). The number of T1D cases is on the rise, with the relative risk for developing the disease ranging from 0.1% [no family history, protective human leukocyte antigen (HLA)] to up to 70% (monozygotic twin with susceptible HLA), and is dependent largely upon genetic susceptibility (1). Importantly, the strongest genetic link to the development of T1D is the expression of certain HLA haplotypes. Class II HLA genes, especially DR3, DR4, and DQ8, are the strongest links; however, HLA Class I molecules also play a role in diabetes development (2, 3). Expression of both Class I and Class II molecules is the largest contributing factor in determining the immune response to a given pathogen, as peptides are processed and presented to T and natural killer (NK) cells *via* the proteins of the major histocompatibility complex (MHC) locus (4). Therefore, these molecules play a key role in directing immune responses, be they beneficial or detrimental. However, the genetic contributions to T1D development are unable to fully account for the increased rates, supporting the idea that environmental factors play a role in the development of T1D. Furthermore, susceptible siblings of T1D patients who are closely monitored frequently show signs of autoimmunity in the form of autoantibodies prior to metabolic dysfunction. Many believe, based on this evidence, that development of full-blown diabetes requires multiple insults to the system in order to manifest itself.

Patients with T1D currently depend upon treatment options that are limited to methods that replace the deficit in insulin production, either *via* injection or transplantation [reviewed in Ref. (5), in press]. While technological advances have helped improve these methods, they still do not provide a cure for the disease. Therefore, determining the mechanisms leading to immune damage of pancreatic beta (β) cells, and treatments to maintain β cell mass, are of the utmost importance.

Recently, perceptions of T1D development have evolved, with a greater attention being paid to islet inflammation as an important event propagating autoimmunity and further loss of β cell mass (6–8). Debates persist as to whether islets are independently inflamed prior to the autoimmune response or the autoimmune response brings about the islet inflammation. One of these recent studies described the incorrect processing of the insulin protein that led to the generation of abnormal peptides recognized by circulating CD8^+^ T cells in T1D patients (8). This line of evidence certainly points to β cell defects contributing to diabetes pathogenesis; however, this study does not address what might cause β cells to produce this incorrectly processed protein. One study in non-obese diabetic (NOD) mice has suggested that incorrect protein processing in these mice causes an increase in endoplasmic reticulum (ER) stress, and results in the development of autoimmunity (9). Given the lack of complete concordance among monozygotic twins, many believe external environmental factors, such as viruses, strongly influence the development of islet inflammation leading to T1D. Trying to understand all of these data in concert brings researchers in the field to ponder the chicken and egg scenario. Are either islets or immune cells in susceptible individuals causing the initial insults that spark diabetes development, or does an environmental factor trigger the disease? Do we see signs of virus infections in patients with T1D because the infection is what precipitates diabetes development, or are patients with diabetes more susceptible to developing virus infections because of defects in their bodies’ defense systems? With the data that are currently available, the order of events in the precipitation of T1D is unclear.

## A New Hypothesis

As we gather more evidence, it is becoming clear that we must look at the integrated physiology to fully understand the mechanism(s) of T1D development. Here, we outline an idea that incorporates early antiviral immune effectors, NK cells, with proinflammatory processes involving 12-lipoxygenase (12-LO) occurring in the pancreatic beta cells. We hypothesize that the activation of NK cell 12/15-LO (*Alox15*, in mice) or 12-LO (*ALOX12*, in humans) through environmental triggers, such as Coxsackievirus infection, contributes to T1D initiation by affecting the normal innate immune interplay between NK cells and islets, which primes downstream autoimmune responses leading to islet destruction. This may occur, in part, due to the effects of inflammation (including 12-LO) on the balance of NK cell receptor expression (10). Below, we will describe the evidence supporting this hypothesis, beginning with one of the suspected environmental triggers, enteroviruses.

## Direct Evidence for Virus Infections in T1D

Over the past fifty years, there has been accumulating evidence linking viruses, and the patients’ responses to these viruses, to the initiation of T1D. This idea that viruses contribute to the initiation and development of T1D was first introduced in the 1960s (11, 12). This is, of course, difficult to fully pinpoint, as the infection may occur long before disease onset, and scientists with access to human pancreas tissues are granted only a snapshot of the patient’s final day as their window into the disease process. Additionally, as mentioned previously, it is unclear whether or not patients susceptible to developing T1D are also more susceptible to developing virus-mediated infections in the pancreatic islets, which might increase the viral signature in the islets of patients with T1D. Therefore, this might not be a causal relationship, but merely coincidental. Since we cannot directly test whether viruses initiate T1D in humans, researchers have used animal models to test this theory. NOD mouse models have been used to show that Coxsackievirus B1 and B4 (CVB1, CVB4) infection speeds diabetes pathogenesis (13), and is dependent upon host sensors of virus (14–16). Others have shown that these effects are highly age dependent, as infection at before 10 weeks of age can prevent diabetes development (17, 18). Additionally, studies of immunodeficient mice engrafted with human islets have shown that human islets can become infected with CVB4, which causes direct damage to the β cells, and results in diabetes. Gene expression profiles of these infected islets indicated significant increases in genes related to the Type 1 interferon (T1-IFN) pathway, as well as genes related to ER stress (19). While these data support the idea of viruses contributing to diabetes development, they do not answer the question about which occurs first: islet dysfunction or immune activation.

To address the role of virus infections in human T1D, groups such as Persistent Virus Infection in Diabetes Network and the Network for Pancreatic Organ Donors with Diabetes—Viral Working Group (nPOD-V) have approached the question with great coordination across multiple platforms (20) assessing the same donor samples (PCR, immunohistochemistry, proteomics, and ISH). These team science efforts have yielded results estimating that Coxsackievirus infections might contribute to diabetes development in over 50% of cases (21, 22). While certainly not causal, pancreas tissues, and specifically β cells, from T1D donors have been found to express viral VP1 proteins more frequently than non-diabetic (ND) donors (23, 24). These studies continue to progress, generating a wealth of information from human donor samples.

Mechanistically, enteroviruses can infect *via* a fecal/oral route, thereby implicating intestinal involvement during the infection process. Mounting evidence has shown a role for the gut microbiome as a contributing factor in autoimmune diabetes development. Viruses and microbiota are known to interact with one another, and shape the response of both parties, which may influence the development of T1D (25). Recent human studies of closely matched control and T1D experimental groups demonstrate both increased inflammation in the duodenum of T1D patients (26), and direct detection of enteroviruses (27). In the first study, donors were tested for markers of inflammation using histological techniques and PCR array, indicating significant inflammatory processes in T1D donors, including increased macrophage numbers in the duodenum of T1D (26). In the second study, T1D donors were much more likely to have markers of enterovirus infection than control donors independent of HLA haplotypes, as tested by *in situ* hybridization and histological techniques (27). This work could not conclude whether T1D patients were more susceptible to the virus infections, or the infections are persistent. Coxsackievirus infection of β cells with strains B1 (28) and B4 (13) may occur *via* β cell expression of the Coxsackie Adenovirus Receptor (29, 30) following viral migration from the duodenum to the pancreas through the common bile duct or affiliated vessels (27). Pursuant to the role of islet inflammation following environmental insult, *in vitro* studies indicate that infection could lead to ER stress in β cells, contributing to islet dysfunction that activates the autoimmune response (9, 31). Alternatively, the infection could also directly activate immune responses that become uncontrolled due to inherent immune defects. Until imaging of live T1D patients affords the ability to detect virus infection in real-time, other experimental avenues must be explored, including the use of cultured islets and mouse models.

## Indirect Evidence for Virus Infections in T1D

While viruses themselves may be difficult to detect in our snapshot views of human T1D, there is ample “circumstantial” evidence that exists in the form of immune cells and mediators. Both mouse models and organ donors with T1D have provided clear evidence that islet inflammation is a key hallmark of this disease. Immune cells infiltrate the islets, albeit at different intensities, in both species. Many patients show signs of adaptive immunity against the pancreatic islets in the form of autoantibodies and islet-specific T cell clones.

Beyond cellular responses, cytokines and chemokines also contribute to islet demise and can stem from both innate and adaptive responses. T1-IFNs have recently gained more respect as effectors in the development of T1D (see the review by Newby and Mathews in this issue). Indeed, virus infections are strong stimulators of T1-IFN production, which leads to a subsequent upregulation of MHC Class I expression, another hallmark of T1D (32).

Downstream of this response, numerous proinflammatory cytokines and chemokines have been detected in patients with diabetes (33, 34). One of these, IFN-gamma (IFN-γ), has been shown to play an important, albeit controversial, role in T1D pathogenesis (7, 35). While absence of the cytokine itself leads to delayed disease development (36), absence of the receptor protects against the development of insulitis (37). IFN-γ has many points at which it can act in the development of T1D, from altering MHC/HLA expression on involved cells to altering endothelial cell function and signaling to immune cells to activate cytotoxic effectors (37). Diminished IFN-γ responses can prevent the recruitment of insulitic T cells, as well as their ability to respond to antigens, which might prevent diabetes progression. However, increased IFN-γ production by CD4^+^ T cells can actually contribute to the resolution of CD8^+^ T cell responses (35). While it is appreciated that CD4^+^ T cells contribute to the IFN-γ production during T1D pathogenesis, this does not exclude the idea that NK cells may be the first producers of IFN-γ present in the islets. Interestingly, IFN-γ is also frequently detected following virus infections, and is used by the immune system to combat viral replication. Given these data, it is unclear whether IFN-γ is serving in a proinflammatory capacity or an unsuccessful attempt at tolerance induction during the development of T1D (35).

## NK Cells and Their Role in T1D

Natural killer cells are large granular lymphocytes that are considered part of the innate immune system. While they do not react as quickly as neutrophils and macrophages against invading pathogens, they mount a response more quickly than do T cells from the adaptive arm of the immune system. NK cells are known as key players in fighting off both tumor cells and virus-infected cells. Despite their small number (only 5–10% of leukocytes in the spleen and 1–6% in peripheral blood) (38), NK cells are powerful cytolytic effectors. Upon stimulation by a variety of cytokines, including T1-IFNs and IL-12 (39), NK cells utilize several different mechanisms to lyse their targets: the combination of perforin and granzymes, signaling through death receptors (i.e., Fas/FasL), and antibody-dependent cellular cytotoxicity leading to either apoptosis or necrosis (40). NK cells can also produce potent cytokines, such as IFN-γ and TNF-α (41). IFN-γ production by NK cells might also serve an antigen presenting capacity (42–44), which, along with their potent cytokine production abilities, would give them the power to stimulate immune responses downstream of their own activation.

In order to recognize their targets, NK cells have developed an intricate system of check and balances. As NK cells are expected to determine aberrant “self” cells (tumors and virus-infected cells), they must be able to distinguish which cells are healthy, and which are not. NK cells respond to virus infections in both mouse and man (4) through signaling mechanisms involving a delicate balance of inhibitory and stimulatory receptors expressed by NK cells. Normal expression of MHC Class I molecules (HLA in humans) send “self” signals to NK cells, inhibiting lytic responses (45). Virus infection can lead to the downregulation of MHC Class I molecules on the surface of infected cells. While this prevents CD8^+^ T cells from responding to viral peptides, it also diminishes the inhibitory signal transmitted to NK cells [reviewed in Ref. (46)]. Subsequently, stimulatory signals to the NK cell are able to override inhibitory signals, leading to lysis of affected cells. In some instances, including during infection of pancreatic islets, viruses push the cellular machinery into overdrive and promote Type 1 IFN production (47), causing hyperexpression of the MHC Class I molecules (32). To circumvent this tactic, NK cells utilize receptors that recognize the upregulation of ligands for the natural cytotoxicity receptors, like NKp46, on the surface of infected cells (48, 49). Thus, NK cells can become “licensed to kill” through several mechanisms that allow them to detect alterations in MHC Class I molecules, as well as increased expression of stimulatory ligands, making them versatile effectors during virus infections (50).

Typically, T1D is thought to be dominated by autoimmune T cell responses; however, growing evidence suggests that NK cells are also involved (51). NK cells take up residence throughout the body (52), providing immune surveillance and protection against viruses wherever they enter the body. NK cells are plentiful in the intestines as compared to other organs (53), comprising 20–40% of Intestinal Epithelial Lymphocytes in healthy children (54), as compared to about 10% of the blood and spleen. This provides NK cells ample opportunity to respond to Coxsackievirus infections, as well as others (*Salmonella, Toxoplasma gondii*, other parasites, viruses, and bacteria) (55), transmitted *via* the fecal/oral route. Paired with the evidence of increased inflammation in duodenum of T1D patients, these data support our hypothesis. Furthermore, NK cells have been detected in the pancreas of both diabetic mice and humans. In mice, the cells appear shortly after macrophages (56). In human pancreatic samples, although NK cells are not frequently detected (57), they have been found in insulitic lesions, and show indications of having a protective effect (58). When one considers the frequency of NK cells in lymphocyte-rich organs (5–10% of leukocytes in the spleen), and also accounts for the number of cells required to define insulitis in humans [six or more CD3^+^ cells in at least three islets (59)], then perhaps it is not surprising that NK cells are rarely detected in donor samples. Alternatively, it is possible that NK cells may prime the pancreatic environment for the entry of diabetogenic T cells, and subsequently depart. As we only have access to one time point for each human donor, we cannot currently distinguish these hypotheses. However, the use of mouse models made aid in this differentiation.

Natural killer cells themselves have recently been directly implicated in the development of T1D through additional expression quantitative trait loci analysis following genome-wide association studies, which further suggests that NK cells play a key role in T1D pathogenesis (60). Interestingly, this study indicates that NK cells may impact T1D development more than CD8^+^ T cells. The carefully designed and executed study is limited to only 105 Japanese subjects, which might not apply to other ethnic backgrounds. However, it is comprehensive, and provides a solid approach for other ethnic backgrounds to be tested. Another data set investigating NK cell phenotypes from patients with T1D showed that NK cells from these patients express significantly reduced levels of activating receptors on their surface as compared to healthy controls (10).

It is unlikely that NK cells act independently in T1D development. Macrophages recognize environmental signals, and have been shown to enter pancreatic islets at 3–4 weeks of age in NOD mice (61, 62). Macrophage production of IL-12 and IL-18 can strongly activate NK cells, which are found in the pancreas of diabetes-prone NOD mice as early as 4 weeks of age (56, 63), to produce high levels of IFN-γ (64). Indeed, serum levels of both IL-12 and IL-18 are higher patients with T1D (65, 66), and IL-18 has been shown to participate in T1D pathogenesis of NOD mice (67). Despite a defect in IL-15 signaling in NOD mice (68), which affects NK cell development and function, others have shown in IL-15-deficient mice that increased IL-12 signaling may allow NK cells to overcome this deficit when faced with pathogenic stimuli (69, 70). The early appearance of NK cells in the pancreas may enable them to activate diabetogenic T cell responses.

Perhaps most importantly, NK cells can directly interact with pancreatic islets through expression of ligands for the NK activating receptors NKG2D and NKp46. Both of these receptors have been implicated in NK-mediated self-aggression in human NK cells that can be triggered by signaling through NKG2D and NKp46 (71). The NKG2D ligand, RAE-1 (72), is one of these ligands. Some speculate that NKG2D ligands cause down-modulation of the receptors, thereby making the NK cells less active (73); however, others have failed to validate this hypothesis (74). They instead showed that differential expression of NKG2D ligands did not hinder NK cytotoxicity through methodical assessment of receptor and ligand levels using genetic tools to dictate the alteration of expression.

Pancreatic islets also broadly express ligands for the NKp46 natural cytotoxicity receptor. NKp46 is a Type I transmembrane protein with two extracellular Ig-like domains followed by a short stalk region, a transmembrane domain containing a positively charged amino acid residue, and a short cytoplasmic tail (75, 76). However, the cellular ligands for NKp46 have not been identified or characterized. The only NKp46 ligands identified so far are the hemagglutinin of influenza virus and the hemagglutinin-neuraminidase of parainfluenza virus (48), suggesting a role for sugars in NKp46 ligand recognition. Studies of NKp46 ligands have utilized the NKp46 Fc chimeric protein in flow cytometry and histological techniques to examine expression over time in the islets of mice and humans (63). Functional studies from the same group showed that blockade of NKp46 receptor/ligand interactions protects against streptozotocin-induced diabetes (63). Although these studies have assessed expression over time, differential expression of the NKp46 ligands in human ND controls versus T1D donor samples has not been tested.

**Figure 1:**
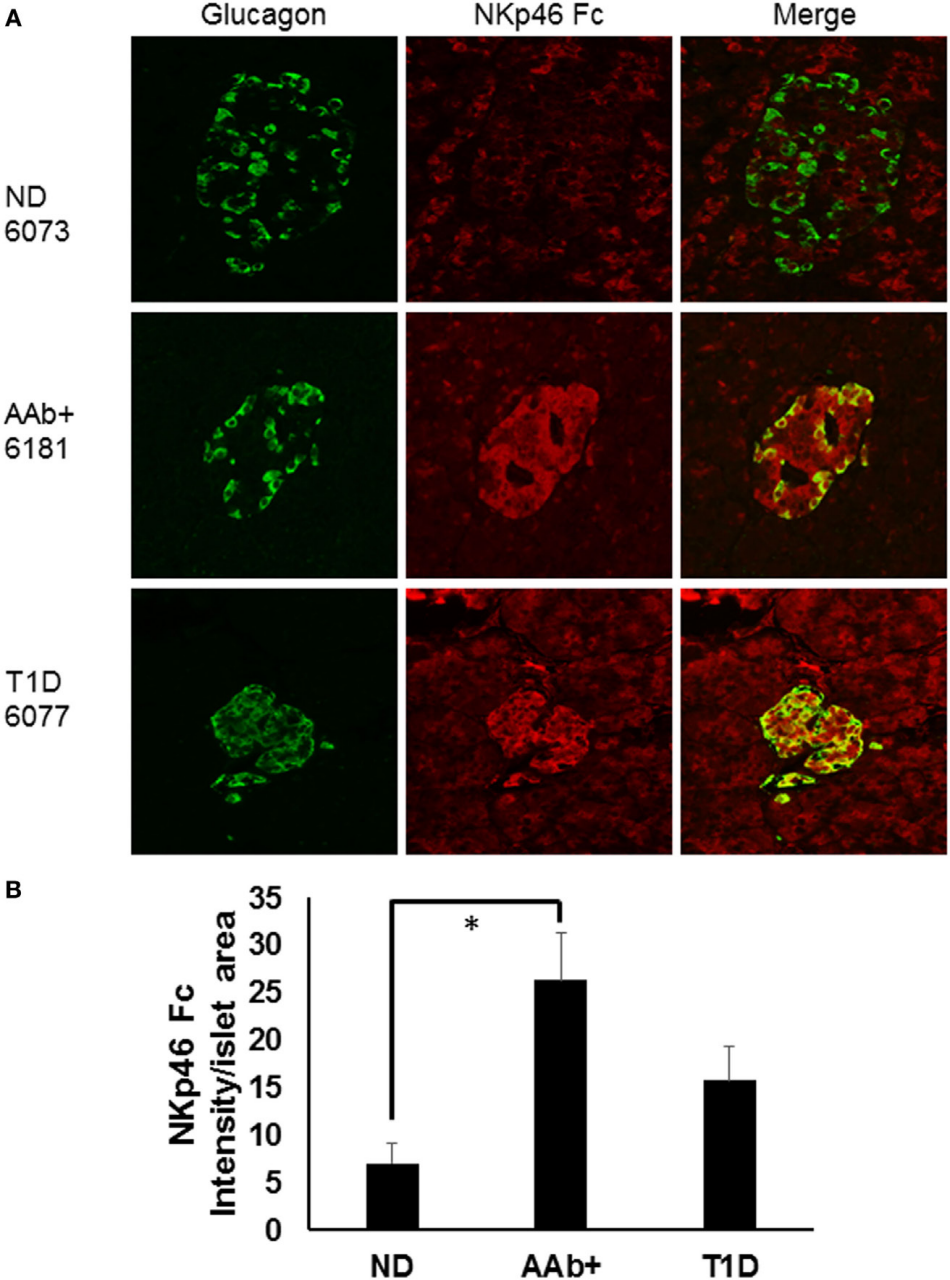
Human islets express NKp46 *ligands*. **(A)** Representative images comparing donor pancreas tissues from Network for Pancreatic Organ Donors with Diabetes (nPOD) biorepository samples. Sections were stained with antibodies against glucagon (green) and the NKp46 Fc chimeric receptor (red). **(B)** Quantification of NKp46 Fc staining per islet area. Density of NKp46 Fc staining was determined for each islet area. Islets for each donor were assessed, and donors of the same group were averaged. *N* = 2 for non-diabetic (ND); *N* = 3 for both autoantibody positive (AAb+) and T1D. *p < 0.05 by one-way ANOVA.

We recently studied ND, autoantibody positive (AAb+), and T1D donor samples from nPOD in order to determine whether there were expression differences in NKp46 ligands using the NKp46 Fc chimeric protein. Figure 1A highlights representative images from donors with different health statuses. Islet images from two ND, three AAb+, and three T1D donors were analyzed by NIH Image J to quantify the density of NKp46 Fc staining (red) within the islet area as determined by glucagon staining (Figure 1B). Islets from T1D donors frequently retain alpha cell mass longer than insulin-positive beta cell mass (77, 78). Therefore, we used glucagon staining to more accurately determine the islet area for each donor in order to calculate the intensity of NKp46 Fc staining in islets. Interestingly, NKp46 Fc levels were significantly higher in the AAb+ donors as compared to ND donors. Although the difference was not statistically significant when comparing T1D and AAb+ donors, there was a trend toward higher expression in the AAb+ donors. These data suggest that NKp46 ligands are upregulated during the development of T1D, and diminish as the islet health and mass decrease over the course of the disease. While this may be the result of ongoing immune responses in these tissues, insulitis has only been detected in one each of the AAb+ and T1D donors. General characteristics of donors tested are listed in Table 1.

**Table 1:** Donor profiles for NKp46 ligand staining.

nPOD case #	Donor type	Age	AAb+	Diabetes duration (years)	Insulitis
6048	ND	30	—	—	N
6073	ND	19.2	—	—	N
6151	AAb+	30	GADA	—	N
6181	AAb+	31.9	GADA	—	N
6197	AAb+	22	GADA, IA2A	—	Y
6077	T1D	32.9	mIAA	18	N
6083	T1D	15.2	mIAA	11	N
6088	T1D	31.2	mIAA, GADA, IA2A, ZnT8	5	Y

Mechanisms of NK cell action in diabetes are not well understood, and phenotypic differences in NK cells residing in different tissues may confound the results reported to date (40). Increased expression of stimulatory receptors on NK cells has been reported in both diabetic mice (79) and humans (80), while others maintain that a lack of NK cell activation contributes to diabetes development (10, 68, 73, 81). This could be due in part to the type of analysis, as genomic studies do not always translate to protein expression. Several groups have studied NK cells in NOD mice. Both found that murine pancreatic NK cells exhibit a different phenotype from those found in the spleen and lymph nodes and have increased proliferative capacity (56, 82); however, there is not a consensus on levels of IFN-γ production, as one indicates lower levels *ex vivo*, but normal levels *in vivo* (82). Depletion of NK1.1^+^ cells in NOD.NK1.1 congenic mice did not significantly affect disease onset, but the depletion protocol also removed NK/T cells (82). These tissue-specific phenotypic differences may also alter the detectability of the NK cells residing within the human pancreas using standard methodologies, but this has not yet been studied in humans. NOD mice have been shown to have a defect in IL-15 production, which contributes to NK cell dysfunction (68). IL-15 is required for NK cell maturation, and although NOD mice are not completely IL-15 deficient, they do show impaired NK cell development. Taken in concert with other systemic alterations in the NOD strain, the impact of this IL-15 defect has not been fully explored. As mentioned previously, NK cells functionally adjust to the absence of IL-15 by responding to IL-12 and IL-18 in order to produce IFN-γ (83), and by responding to pathogenic stimuli in the presence of IL-12 (69).

## The Proinflammatory Mediator, 12-LO, in T1D

12-Lipoxygenase [(12-LO) gene name *ALOX12S* in humans; 12/15-lipoxygenase (12/15-LO), gene name *Alox15* in mice] converts arachidonic acid to the proinflammatory 12(S)-hydroxyeicosatetraenoic acid (12(S)-HETE) through a 12-S-hydroperoxy-eicosatetraenoic acid (12-HPETE) intermediate (84, 85). IL-12 signaling downstream of 12(S)-HETE production (86, 87) activates STAT4 [reviewed in Ref. (88)], contributing to additional inflammation. IL-12 signaling through STAT4 strongly activates NK cells and T cells and is also known to be a strong contributor to autoimmune conditions in general [reviewed in Refs. (89, 90)]. In addition to T1D, 12/15-LO has been implicated in many inflammatory processes, including cancers (91, 92), asthma (93), and Type 2 diabetes (94).

Several lines of evidence indicate a critical role for 12/15-LO in the pathogenesis of T1D. It has been shown that deletion of STAT4 signaling molecules, which are downstream of 12/15-LO activation, in NOD mice protects the NOD strain from developing T1D (95). Subsequently, we published that the NOD mouse line congenic for the global *Alox15* (NOD-*Alox15*^*null*^) deletion is >98% protected from developing spontaneous T1D (61). This line boasts a narrow congenic region delineated through extensive microsatellite mapping, and shows significantly reduced disease incidence (~2%). To understand the origin of the protection seen in these mice, wild-type NOD mice were tested for their expression 12/15-LO in islets, macrophages, and lymphocytes. Islets and macrophages expressed the enzyme in appreciable amounts, while lymphocytes had either low or undetectable amounts (96). Additionally, *Alox15*^*null*^ mice have been shown to express decreased levels of IL-12 (96) and IL-18 (97), which are cytokines that contribute to NK cell IFN-γ production (83). Since publication, this strain has been shipped to additional vivaria and maintained this phenotype. Subsequent studies of human islets have also indicated 12-LO expression under inflamed conditions (98, 99), which feeds into the detrimental cycle of inflammation.

**Figure 2:**
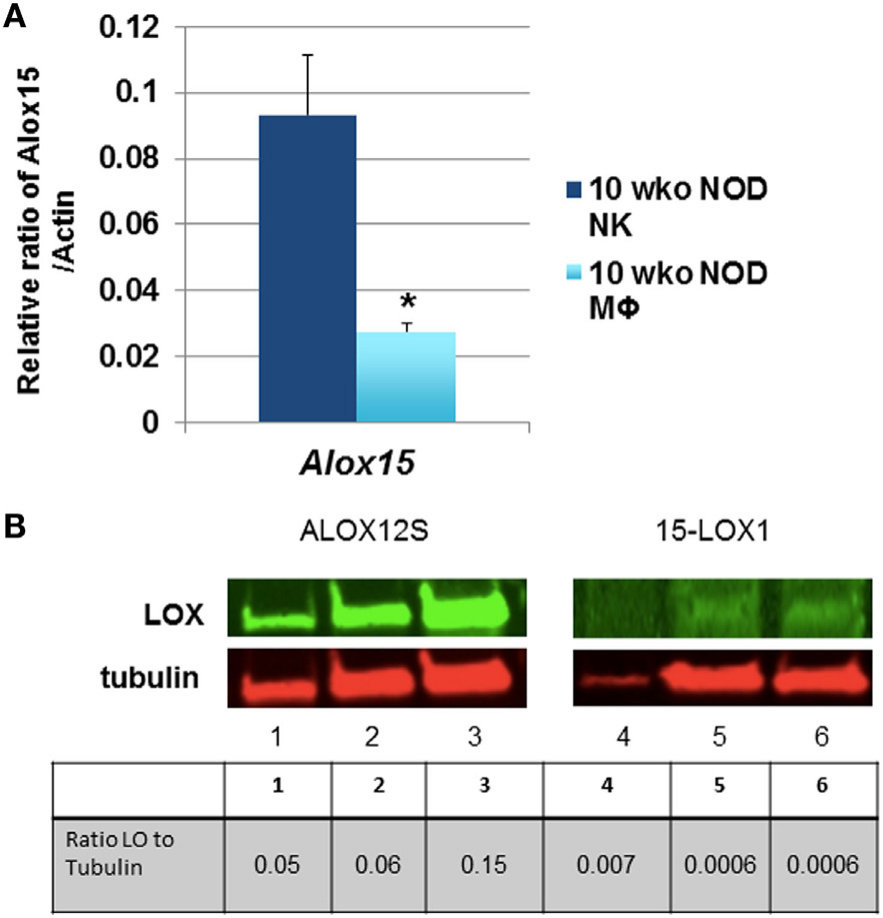
Natural killer (NK) cells express 12/15-lipoxygenase (12/15-LO). **(A)** Murine non-obese diabetic (NOD) natural killer cells express Alox15. mRNA levels of the *Alox15* gene were tested in NK cells from NOD mice. These levels were compared to thioglycollate-induced peritoneal macrophages from 10-week-old NOD mice using the relative ratio of *Alox15/Actb*. *p < 0.05 using a two-tailed Student’s *t*-test to compare NK vs. macrophages in age-matched NOD mice, *n* = 6 mice per group. **(B)** 12-Lipoxygenase (12-LO) protein expression in human NK92 cells. The human NK92 cell line was tested for protein expression of 12-LO by western blotting. ALOX12S expression was most abundant in the cell line, which is the most abundant form found in human islets. Lanes 1 and 4 are nuclear proteins from the two pooled NK92 samples; lanes 2 and 5 are cytoplasmic proteins from one NK92 sample; lanes 3 and 6 are cytoplasmic proteins from different NK92 samples.

**Figure 3:**
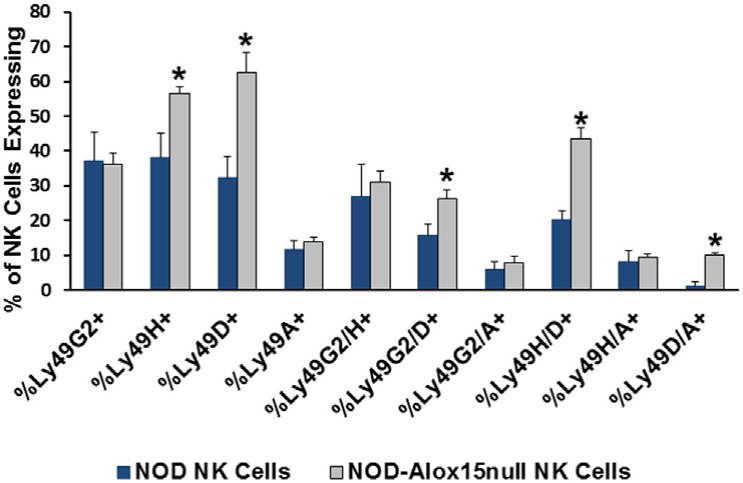
Pancreatic lymph node natural killer (NK) cell expression of NK cell markers. Ly49 receptors determine which targets NK cells recognize and respond to during interactions with potential targets. Several of the expression patterns are altered in the absence of *Alox15*, with *Alox15^null^* cells expressing higher proportions of the activating Ly49 receptors [*p < 0.05 using a two-tailed Student’s *t*-test to compare receptor expression in non-obese diabetic (NOD) vs. NOD-*Alox15^null^* NK cells for each receptor or receptor pair]. *n* = 4 mice/group.

Natural killer cell expression of 12/15-LO has not been extensively studied, although historical data suggest that NK cells expressed a member of the lipoxygenase family (100). Using more modern information and methods, we discovered that both mouse and the human NK92 cell line express 12/15-LO and 12-LO, respectively (Figure 2). Surprisingly, this expression of 12/15-LO in freshly isolated NOD mouse NK cells is significantly higher (3.3-fold more) than that seen in thioglycollate-induced NOD macrophages (61, 96). Since NK cells are closely related to T cells, we expected that expression levels would be similar to those seen in T cells, which is almost undetectable (96). While investigating the downstream effects of 12/15-LO expression in our NOD-*Alox15*^*null*^ mice, we found that the pancreatic lymph node NK cells of the NOD-*Alox15*^*null*^ mice had an increased percentage of NK cells expressing activating markers (Figure 3), which are similar phenotypically to peripheral blood NK cells from ND human controls (10). Taken together, it appears that activation of 12/15-LO, which leads to increased IL-12 levels (96), increases the inflammatory nature of the NK cells, presumably through the 12/15-LO pathway. We are currently testing whether NK cells are better able to resolve infections without contributing to the chronic inflammatory milieu in the absence of 12/15-LO.

Several additional lines of evidence suggest that NK cells are strongly influenced by 12/15-LO activity and that they have the capacity to play an important role in the development of diabetes. As mentioned above, activation of 12/15-LO leads to IL-12 production [reviewed in Ref. (94)], which activates the STAT4 signaling cascade that is required for NK cells to respond functionally (101–103), including production of IFN-γ (104, 105). This combination can exacerbate T1D, although IL-12 can also trigger the activation of different cytokine pathways in the absence of IFN-γ (106). The involvement of IL-12 in T1D pathogenesis is not without controversy. Deletion of the IL-12p40 subunit, which can heterodimerize with either IL-12p35 to form IL-12 or the p19 subunit to form IL-23, did not protect against T1D development (107). This may be due to the effect of inhibiting both IL-12 and IL-23 generation simultaneously, although IL-23 had not been discovered at the time these results were published. Subsequently, the same group published work indicating that administration of exogenous IL-12 exacerbated diabetes development (106). Importantly, when key molecules in the 12/15-LO pathway (i.e., 12/15-LO or STAT4), upstream of IL-12, are disrupted in NOD mice, diabetes is prevented (61, 95).

## A New Model of T1D Development

**Figure 4:**
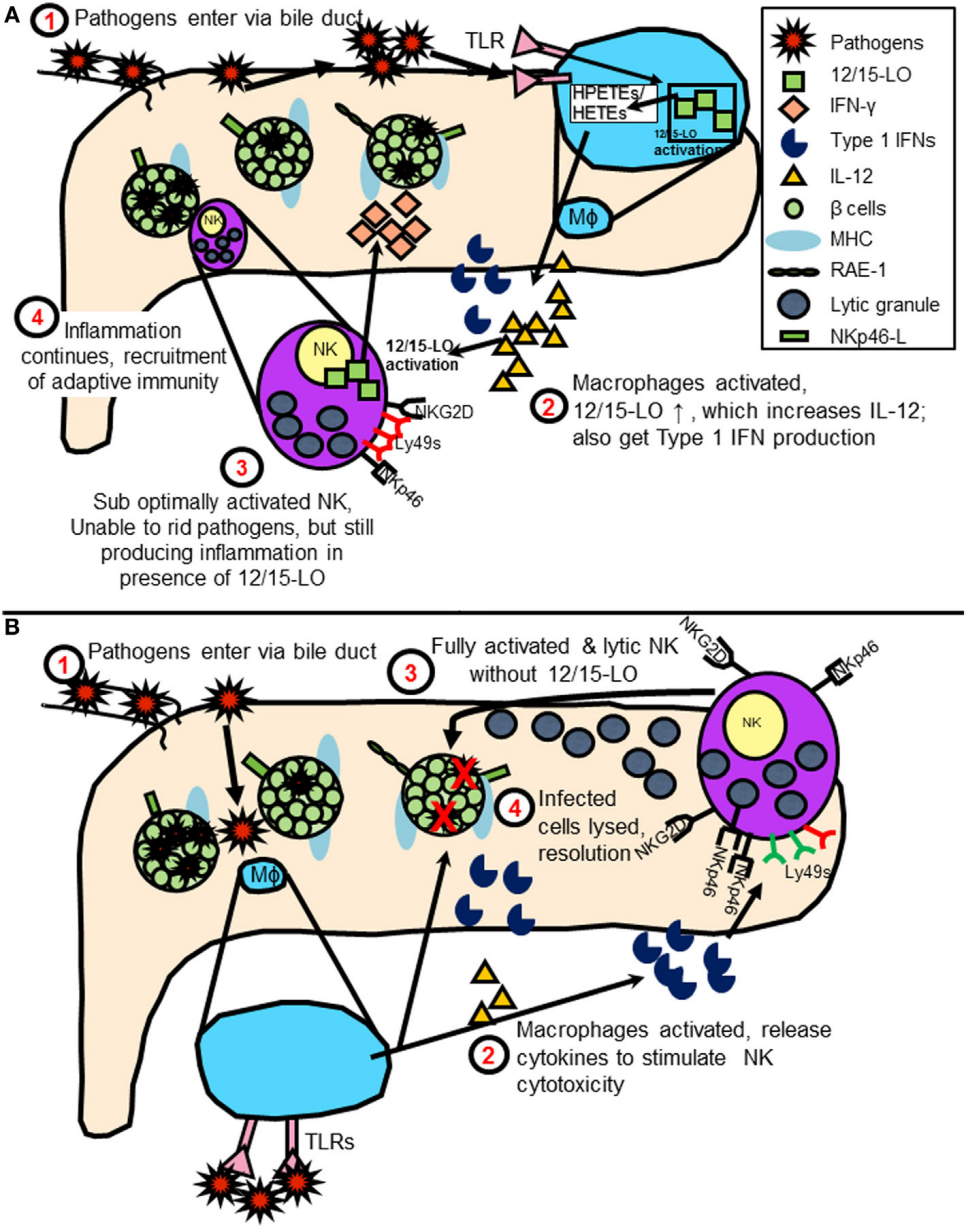
**(A)** Model of natural killer (NK) cell activation leading to type 1 diabetes (T1D) in the presence of 12/15-lipoxygenase (12/15-LO) following virus infection. IL-12 production stimulates NK interferon-gamma (IFN-γ) response, feeds cycle of 12/15-LO activation and inflammation. **(B)** Model of NK cell activation in absence of 12/15-lipoxygenase following virus infection. NK cells respond to macrophage T1 IFN production following TLR signaling by using lytic mechanisms to rid virus-infected cells. Reduced inflammation prevents T1D development.

By bringing NK cell expression of 12/15-LO into the equation of diabetes initiation following virus infection, one can envision a model in which duodenal NK cells encounter some sort of pathogen- or virus-infected cells, such as Coxsackievirus-infected cells. As the pathogen is transmitted through the bile duct or related vasculature on the way to the pancreas, NK cells and macrophages are alerted. Under normal conditions, NK cells and macrophages quickly dispense of the virus, removing only the infected cells. In a patient susceptible to developing diabetes, the interactions are altered, perhaps due to activation of 12/15-LO either by the virus directly or due to increased stress placed upon the beta cells upon infection (19). This leads to abnormal interactions between the innate immune cells with islets expressing NK cell ligands and prevents the resolution of the infection. 12/15-LO activation is known to feed into a vicious cycle of chronic inflammation, which in this instance, may be perpetuated by macrophages and NK cells (shown graphically in Figure 4). As mentioned earlier, in individuals with susceptible HLA haplotypes, or in mice (in was left out inadvertently) with susceptible MHC haplotypes, chronic inflammation signals the diabetogenic T cells to join the fight. This leads to significant islet destruction. Both HLA Class I and II molecules strongly influence the T cell responses in humans, as they dictate the ability of the T cells to recognize and react to autoantigens during the T cell development process, as well as in the periphery (108). This autoreactivity can be precipitated by stress placed on the islets, perhaps due to inflammatory processes. Such stress may lead to HLA Class II-mediated recognition of hybrid insulin (109) or posttranslationally modified (110) peptides by CD4^+^ T cells, thereby breaking peripheral tolerance to neoantigens and furthering the disease progression.

## Conclusion

We have hypothesized that activation of NK cell 12/15-LO (or 12-LO, *ALOX12*, in humans) contributes to T1D initiation by affecting the normal innate immune interplay between NK cells and islets, which primes downstream autoimmune responses leading to islet destruction.

At best, our current understanding of T1D initiation is murky. It is appreciated that there is a role for cells of the mucosal-associated lymphoid tissues, including NK cells, and it is quite likely that infectious initiation of T1D would occur through fecal-oral routes. However, T1D progression also requires the presence of macrophages, which produce 12/15-LO. Following the appearance of the macrophages in the pancreas of NOD mice, NK cells, which also produce 12/15-LO, are found. This process in humans has not yet been delineated, and, therefore, it is unclear if NK cells are migrating from the gut to the pancreas following an infection, or if they are recruited by other means. Macrophages have both the ability to respond to virus infections through TLR signaling, as well as activate NK cells through IL-12 and Type 1 IFN production. It is known that IL-12 production in macrophages is increased following 12/15-LO activation and that IL-12 signaling can feedback into the 12/15-LO signaling cascade (84). NK cell 12/15-LO is then a target for activation following IL-12 stimulation. IL-12, in concert with IL-18, is also known to drive IFN-γ production by NK cells (83). Both IL-12 and IL-18 are increased in mouse models of T1D (67, 96), as well as in patients with T1D (65, 66), and both are increased in the presence of 12/15-LO (96, 97). NK cell-derived IFN-γ could aid in expanding the effector T cell population (111). Conversely, in the absence of 12/15-LO, normal IL-12 levels [as generated through TLR signaling (112)] and Type 1 IFNs from activated macrophages might play a stronger role in influencing pancreatic NK cell function, leading to upregulation of activating receptors, optimal cytotoxic activation, and clearance of viral pathogens with minimal residual inflammation.

Moving forward, it is important to understand mechanisms by which environmental factors might spark the activation of 12-LO in NK cells, macrophages, and islets, leading to the development of T1D. While some pieces of this puzzle remain to be placed, there is striking evidence that our hypothesis and model is possible. Many of the remaining questions can be answered in part by the use of novel global and conditional knock-outs of 12/15-LO on the NOD background in experiments with Coxsackievirus infections. By understanding the order in which these events occur, we will be better able to design selective therapies that might prevent the disease development and progression without resorting to global immunosuppression.

## Methods

### Mice

Female NOD/ShiLtJ (NOD) mice were ordered from Jackson Laboratory (Bar Harbor, ME, USA); global NOD-*Alox15^null^* mice (bred on-site at EVMS) were housed in SPF conditions and treated in accordance with the AAALAC and IACUC guidelines at the Eastern Virginia Medical Center. Mice were euthanized by asphyxiation with CO_2_. Blood glucose levels were assessed following euthanasia at 4 and 10 weeks of age. Spleens, lymph nodes, and islets were removed.

### Cell Isolations

Natural killer cells were isolated from spleen using cell isolation kits from Stem Cell Technologies (Vancouver, BC, Canada) per the manufacturer’s instructions. Purity of the isolated populations was assessed by flow cytometry (see below) after staining with antibodies against cell surface markers including anti-CD3, anti-CD19, anti-NKp46, and anti-CD11b. Cells were generally 85–90% pure.

### Flow Cytometry

Cells isolated from the pancreatic draining lymph nodes of NOD and NOD-*Alox15*^*null*^ mice at 10 weeks of age were stained with antibodies against cell surface markers for T (CD3), B (CD19), and NK cells (NKp46, Ly49A, Ly49G2, Ly49D, Ly49H). Gates were determined by using fluorescence minus one controls. All antibodies were purchased from Biolegend (San Diego, CA, USA).

### qRT-PCR

mRNA was isolated from indicated cells and tissues using the RNeasy Kit from QIAgen (Germantown, MD, USA), and used to generate cDNA for use in qRT-PCR assays as described (96). 12/15-LO expression in mouse cells was assessed by a SYBR green protocol, and compared to a newly available *Alox15* Taqman probe (Thermofisher Scientific, Waltham, MA, USA). Expression was tested in two independent experiments using five mice per group for each experiment.

### Western Blotting

Cell lysates from the NK92 human NK cell line was used as a source of proteins to measure level human 12-LO levels using the Odyssey LI-COR system (Lincoln, NE, USA) as previously described (61). Nuclear and cytosolic proteins were fractionated and tested separately. Duplicate samples were stained with antibodies recognizing tubulin, ALOX12S, and ALOX15-1.

### Immunofluorescence

Formalin-fixed, paraffin-embedded tissues sections from human pancreas tissues (obtained through nPOD) were stained as described with antibodies to glucagon (DAKO, Copenhagen, Denmark), and the NKp46 Ligand (using the NKp46 Fc chimera, R&D Systems, Minneapolis, MN, USA) as described previously (48), with a modification using tyramide amplification (Perkin Elmer, Waltham, MA, USA) to amplify NKp46 Fc staining. Islet area was determined, and the intensity of NKp46 Fc staining within the islet area was calculated using NIH Image J. Data are expressed as density of NKp46 Fc staining per islet area for ND, autoantibody positive, and T1D donor (*n* = 3 donors per group), which is calculated with the following equation: [Integrated density − (area of selected cell × mean fluorescence of background readings)]/total islet area = average fluorescence per islet.

### Statistical Procedures

Statistically significant differences were determined by the use of Student’s *t*-test where appropriate, or ANOVA followed by *post hoc* testing. Significant differences in all cases were determined by *p* < 0.05.

## Ethics Statement

This study was carried out in accordance with the recommendations of “Principles of laboratory animal care” (NIH publication no. 85–23), AAALAC, and IACUC guidelines at the Eastern Virginia Medical Center. The protocol was approved by the IACUC at Eastern Virginia Medical Center. The studies of the nPOD human donor tissues were considered to be exempt and deemed “Non-Human Subjects Research” by the Eastern Virginia Medical School Institutional Review Board due to the nature of the donors. All donors are deceased and de-identified.

## Author Contributions

MM conceived the hypothesis outlined. MS and MM wrote the manuscript. MM, LG, and MS contributed to experimental design and data analysis. MS and LG critically reviewed the manuscript prior to submission.

## Conflict of Interest Statement

The authors declare that the research was conducted in the absence of any commercial or financial relationships that could be construed as a potential conflict of interest.
